# Routine measurement of oesophageal temperature during cryoballoon pulmonary vein isolation

**DOI:** 10.1007/s12471-021-01551-0

**Published:** 2021-02-18

**Authors:** H. F. Groenveld, B. A. Mulder, Y. Blaauw

**Affiliations:** Department of Cardiology, Heart Centre, University of Groningen, University Medical Centre Groningen, Groningen, The Netherlands

Dear Editor,

With great interest, we read the recent article by Molenaar et al., in which they presented data on measurement of oesophageal temperatures during pulmonary vein isolation (PVI) using the second-generation cryoballoon [1]. Molenaar et al. included 204 consecutive patients who underwent PVI. Low oesophageal temperature—defined as < 20 °C—was observed in 26% of the patients. A close proximity between the oesophagus and the pulmonary vein was associated with low temperatures. No endoscopic evaluation of development of oesophageal lesions was performed. The authors suggested to routinely use oesophageal temperature measurements during cryoballoon PVI.

Monitoring of the endoluminal oesophageal temperature during atrial fibrillation ablation is performed with the intention to prevent major complications, such as atrio-oesophageal fistula (AOF) or gastroparesis. One of the most feared complications of PVI is the development of AOF. In a recent user-reported survey composed of 500,000 PVIs performed with a cryoballoon, the reported incidence of AOF was 0.004% [2]. Although extremely rare, the related mortality is high (68.8%) [2]. Considering the low incidence, it is challenging to identify risk factors that predict the development of AOF during cryoballoon PVI [3]. The anatomical close proximity of the oesophagus to the vein makes it vulnerable to low temperatures and subsequent oesophageal ulceration during PVI [3]. In patients who were admitted with AOF, most of these fistulas were located near the left inferior pulmonary vein. It was also observed that during cryoballoon PVI, nadir temperature appeared to be of no significant influence; however, longer inflation times did have a significant effect [3].

Measurement of the oesophageal temperature is a controversial subject. In a previous study, patients underwent endoscopy for detection of oesophageal injury following cryoballoon ablation [4]. Oesophageal temperature monitoring showed that lower temperatures were associated with a higher incidence of and more severe oesophageal lesions. However, these lesions were asymptomatic and disappeared within two weeks after ablation [4], which is likely to occur in many patients who undergo atrial fibrillation ablation. Since the lesions have no clinical consequences, it is questionable whether it is necessary to prevent them.

Still, one may argue that these lesions are a precursor of potential AOF and that oesophageal temperature monitoring can prevent this, although there are currently no data available to support this. A previous meta-analysis (including radiofrequency ablation studies) showed that measurement of temperature does not prevent oesophageal lesions [5]. In addition, in a recent trial, patients were randomised to radiofrequency catheter PVI plus oesophageal temperature monitoring versus PVI without monitoring. There was no difference in endoscopically diagnosed oesophageal lesions between the two groups [6]. Considering the higher incidence of oesophageal lesions and AOF (1:1500) when using radiofrequency, a significant difference in oesophageal lesions during cryoballoon PVI with or without monitoring is even less likely [5].

In our opinion, a low oesophageal temperature observed is a surrogate endpoint without proven clinical significance. The currently available research does provide evidence that oesophageal temperature monitoring during PVI cannot prevent oesophageal injury nor AOF. In addition, given the substantial costs of these temperature probes and the very low incidence of these severe complications, we believe that they cannot be recommended for routine use, until the available evidence shows otherwise.
